# Implant-Prosthetic Rehabilitation in Bilateral Agenesis of Maxillary Lateral Incisors with a Mini Split Crest

**DOI:** 10.1155/2016/3591321

**Published:** 2016-04-13

**Authors:** M. M. Figliuzzi, A. Giudice, S. Pileggi, D. Pacifico, M. Marrelli, M. Tatullo, L. Fortunato

**Affiliations:** ^1^Department of Periodontics and Oral Sciences, “Magna Graecia” University, 88100 Catanzaro, Italy; ^2^Biomedical Section, Stem Cells Unit, Tecnologica Research Institute, 88900 Crotone, Italy; ^3^Biomedicine Unit, Calabrodental Clinic, Via E. Fermi, 88900 Crotone, Italy

## Abstract

The reported clinical case describes the surgical procedure of ridge augmentation by using a “split crest” technique with a partial thickness flap and a subsequent implant-prosthetic rehabilitation aimed at treating a bilateral agenesis of the upper lateral incisors. In such cases with vestibule-palatal and mesial-distal scarce bone thicknesses associated with the need of a proper functional and aesthetic rehabilitation, the split crest technique is particularly suitable. In the case we reported, because of the poor bone thicknesses, we performed a minimally invasive split crest which allowed a correct insertion of the fixtures. This technique allowed us to achieve an optimal functional and aesthetic rehabilitation; moreover, we obtained a good emergency profile, ensuring the vitality of the close teeth and ensuring a good primary stability and the following osseointegration of dental implants.

## 1. Introduction

Tooth agenesis is one of the most frequent dental anomalies. Dental agenesis is clinically apparent because of the lack of one or more teeth: for this reason such alteration is one of the best candidates for implant-prosthetic rehabilitation. The literature reports that lateral incisors are affected by agenesis in 2.2% of cases [1].

When the agenesis affects the lateral incisors, besides the functional issues, the greater inconvenience is represented by the aesthetic reasons.

The most suitable solution for these clinical conditions is, of course, an implant-prosthetic rehabilitation, but this surgical approach is not always feasible.

We well know that the critical condition for a good osseointegration is to have an amount of at least 2 mm of healthy bone around the implant [2, 3].

It is also necessary to ensure a good dental emergence profile, to obtain a correct prosthetic rehabilitation from both aesthetic and functional points of view [4].

These conditions are not always possible and the presence of an exiguous bone thickness forces the surgeon to change the axis of insertion of the implants, exposing the prosthetic restoration to the concrete risk of failure.

## 2. Split Crest

During the last years, several methods were offered to solve the problem of scant bone thickness in those cases requiring an implant-prosthetic rehabilitation.

Indeed, thanks to several GBR (Guided Bone Regeneration) strategies, the idea to place the implants where the bone amount is abundant turned into an inclined bone guided arrangement [5].

Many authors proposed the use of bone grafts taking samples from oral tissues (mandibular branch); others preferred instead the extra-oral tissues (e.g., iliac crest bone) [6]. These methods usually led to good results, but they are very aggressive and cannot exclude complications such as additional surgical procedures. To find an alternative solution for increasing the thickness of the crest, a split crest approach between vestibular and lingual cortical layers was proposed, in order to induce new bone formation in the inner area.

The first original study by Osborn is dated 1985: it reported the green-stick fracture technique [7].

This technique was furtherly modified by Bruschi and Scipioni who used partial thickness flaps, obtaining great results [8, 9]. Since the partial flaps preserve periosteum integrity, it allows maintaining the vascular supply of the bone, thus enhancing its repairing capacities. Also hybrid methods were proposed: they coupled the advantages of partial flap approach to synthetic biomaterials use, to extend the high regenerating capacities of the periosteum [10].

## 3. Clinical Case

**Figure 1:**
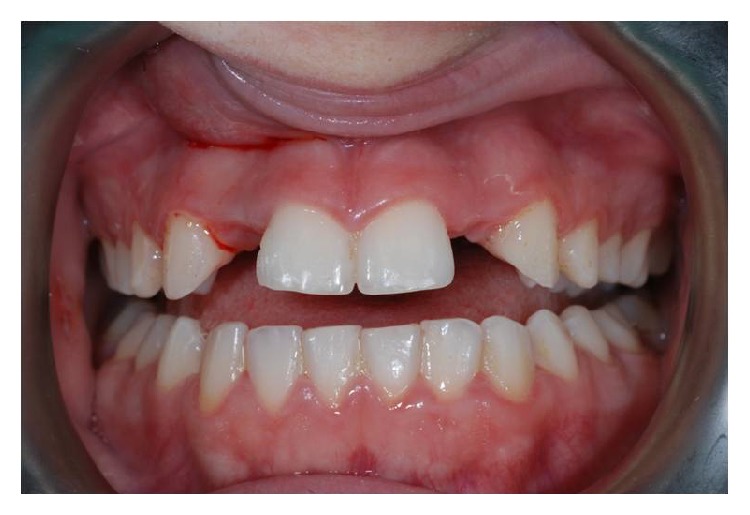
Preoperative clinical condition.

The patient here reported was 26 y/o: she showed a bilateral agenesis of the upper lateral incisors (Figure 1).

The agenesis of teeth 1.2 and 2.2 caused a mesial shifting of the teeth close to the space left empty by the missing elements.

Consequently, an orthodontic treatment was performed to gain the space required to allow the insertion of dental implants. After the orthodontic treatment, a dental “Maryland” bridge was placed as a temporary rehabilitation, in order to ensure the stability of the occlusion, the proper function, and acceptable esthetics [11].

The newly obtained spaces were carefully evaluated to properly plan the implantology in such sites.

The bone showed a thin thickness both in vestibular-palatal and mesial-distal directions.

**Figure 2:**
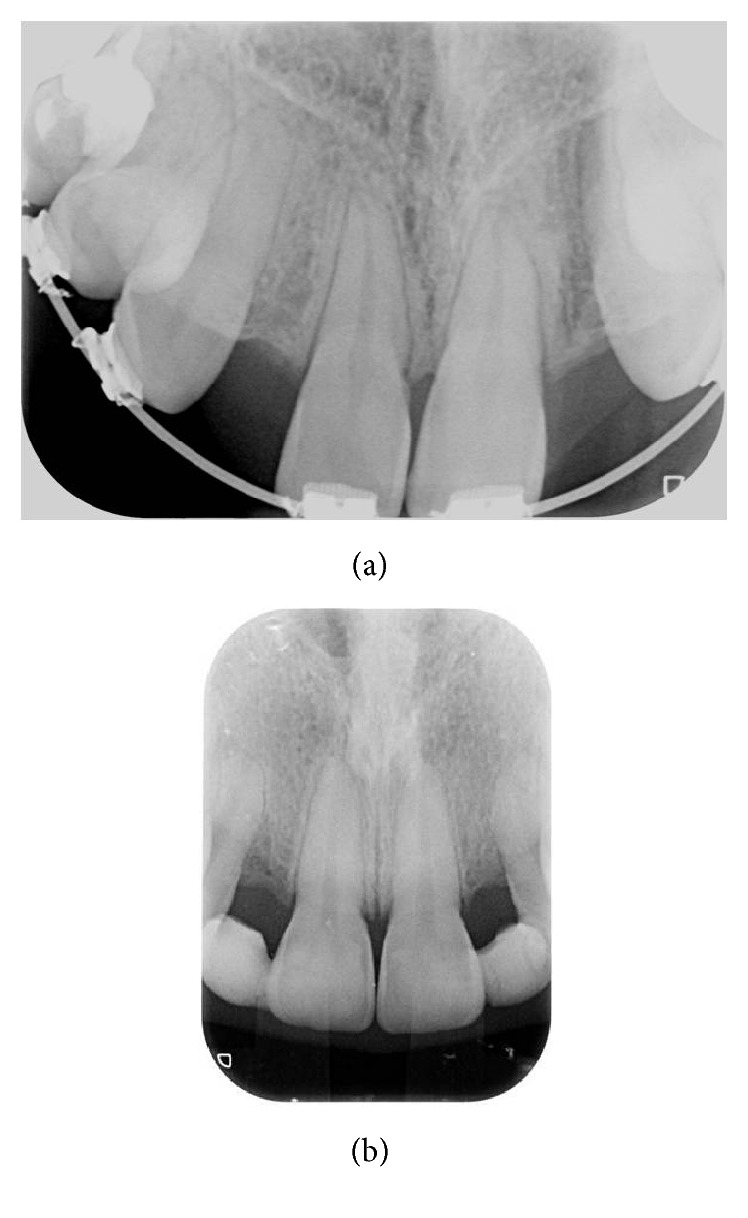
Radiographic preoperative condition.

After oral and extra-oral clinical inspections and radiography examinations, the bone thickness was about 3 mm in crest in vestibule-lingual direction (Figures 2(a) and 2(b)).

The lacking bone depths impeded the right allocation of the implants and thus affected the prognosis.

Therefore, it was decided to proceed with a split crest surgical technique, to gain the necessary space for the implants insertion. For both the sites the procedure was performed as follows:

- local anesthesia,
- incision of the partial thickness flap,
- crestal osteotomy with Beaver 64 blade,
- distraction of vestibular cortical plate,
- preparation of the implant site with the proper burs,
- parallelism verification,
- implant insertion,
- suture of the flap with underlying periosteum anchorage.

## 4. Flap Incision

**Figure 3:**
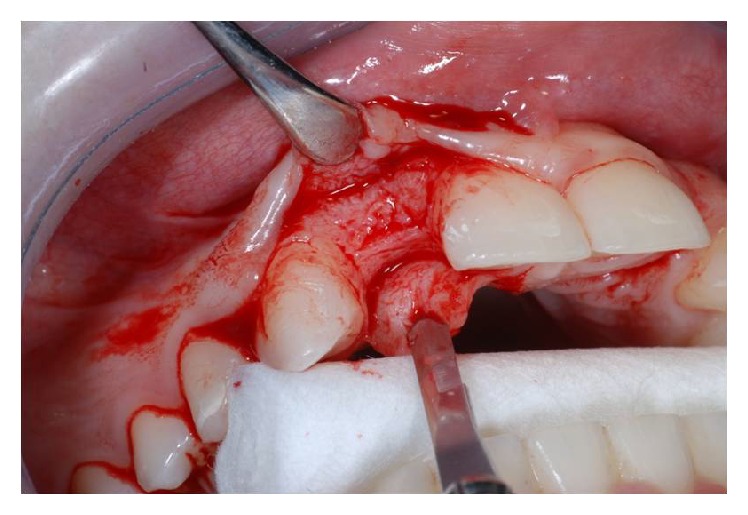
Flap incision in site to the left.

**Figure 4:**
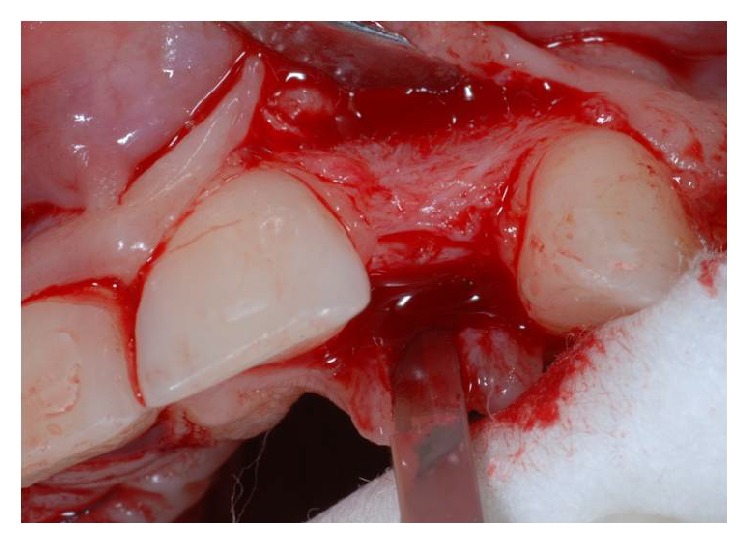
Flap incision in site to the right.

After having anesthetized the surgical site, a partial thickness trapezoidal flap was made through the blade Beaver 64. The incision was performed at the bone crest, with mesial and distal releasing cuttings (Figures 3 and 4).

The cuts were sharp and divergent to ensure an adequate blood supply and therefore an optimal healing.

## 5. Crestal Osteotomy and Bone Window Creation

The Beaver 64 blade was positioned at the top of the crest and was sunk for 4 mm of the cortical bone by a surgical mallet, so as to make a proper bony fracture.

In the same way two other fractures involving mesial-distal release were realized, to mobilize the bone fragment and to shape an operculum. The operculum must have smaller dimensions than the flap to ensure an optimal healing and to avoid dehiscence and infections.

## 6. Osteodistraction

**Figure 5:**
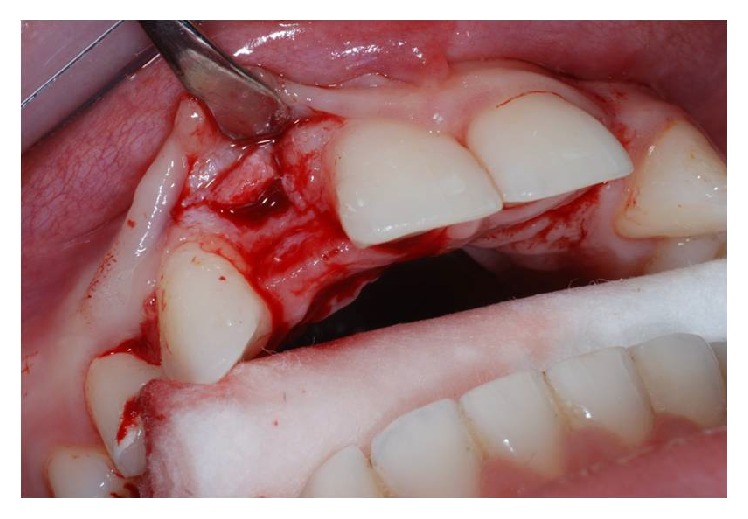
Crestal osteotomy and bone window creation in site to the left.

**Figure 6:**
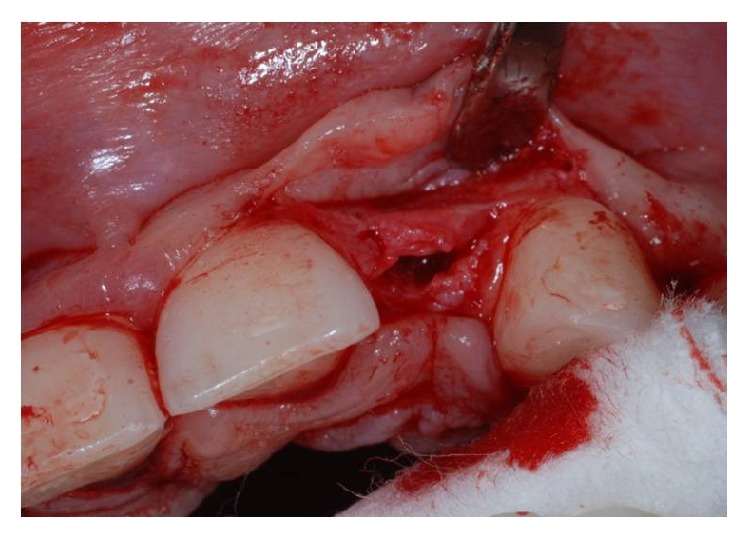
Crestal osteotomy and bone window creation in site to the right.

Using the Bard Parker and then a straight dental elevator, the last bone fragment was displaced (Figures 5 and 6). The surgeons must be careful during this step not to interfere with the apical anchorage of the bone fragment; taking into account this suggestion, the partial thickness flap holds the periosteum on the bone fragment.

## 7. Site Preparation and Implant Insertion

After the dislocation of the bone fragment, we proceeded to the insertion of a series of burs to prepare the implant site.

This operation was performed paying great attention to avoid the risk of injury to the closest dental roots.

**Figure 7:**
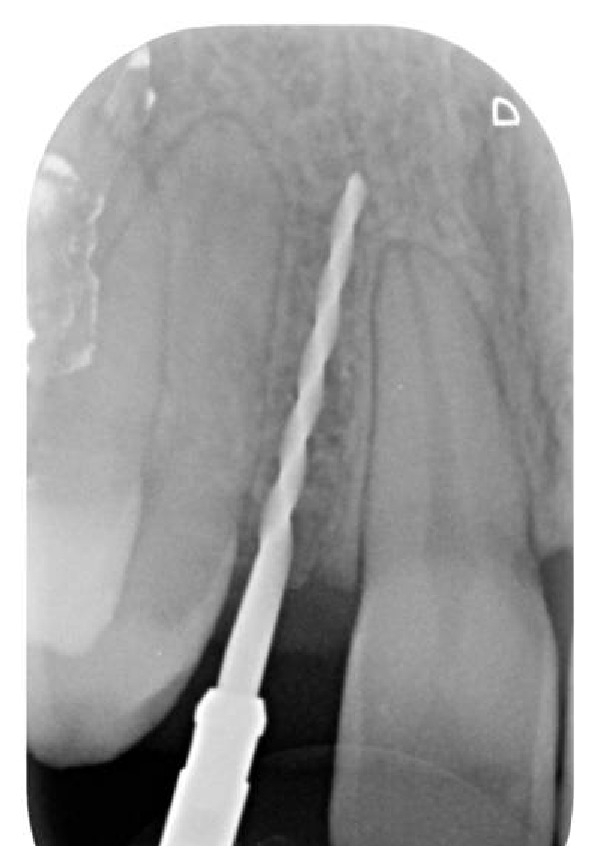
Radiographic examination of the correct inclination in site to the left.

**Figure 8:**
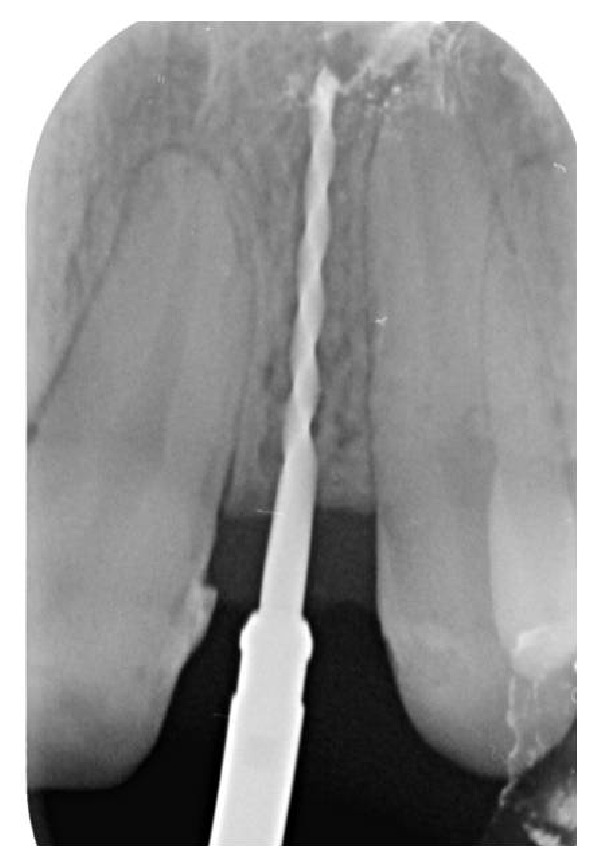
Radiographic examination of the correct inclination in site to the right.

**Figure 9:**
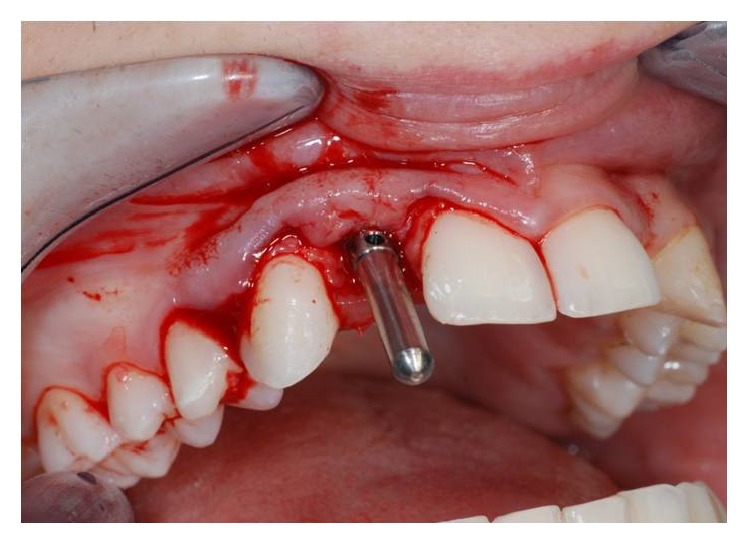
Clinical examination of the correct inclination.

**Figure 10:**
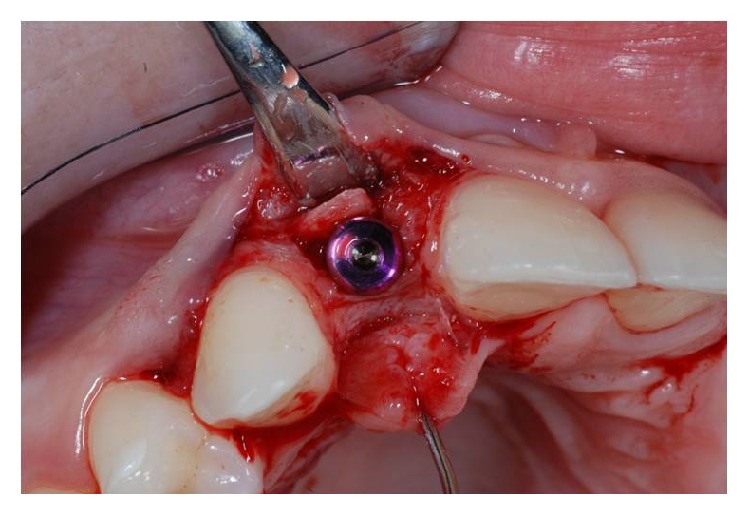
Implant placement in site to the left.

**Figure 11:**
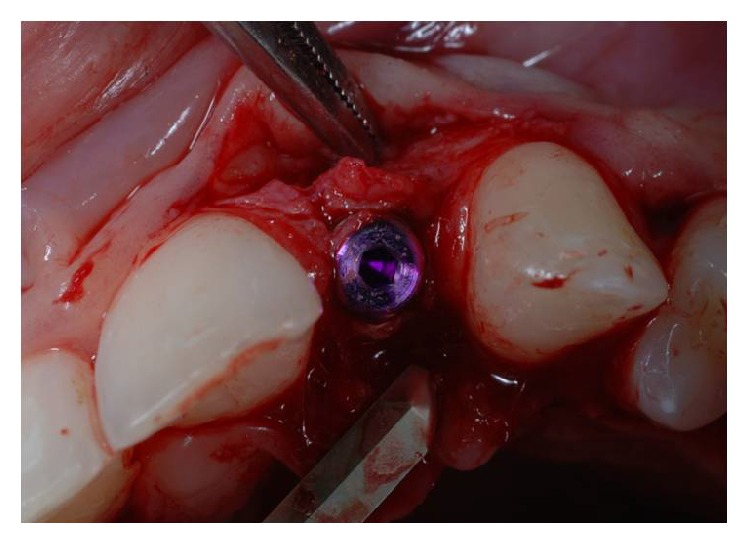
Implant placement in site to the right.

**Figure 12:**
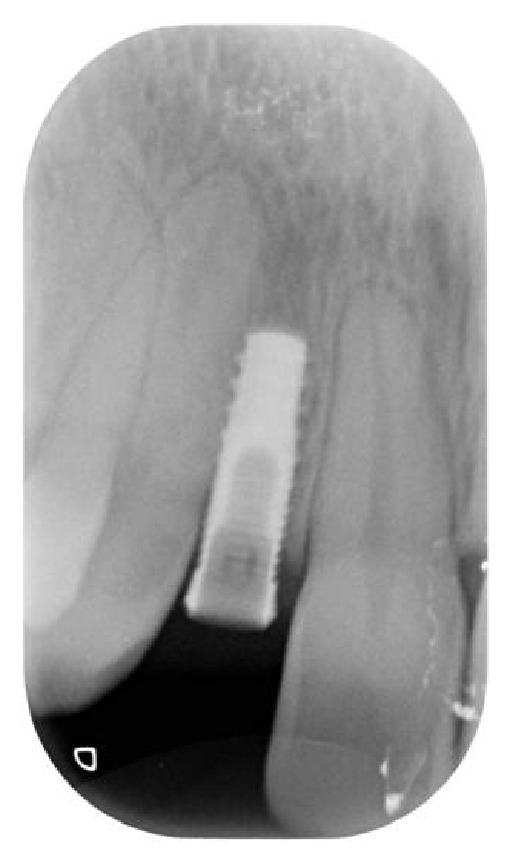
Radiographic control after implant placement in site to the left.

**Figure 13:**
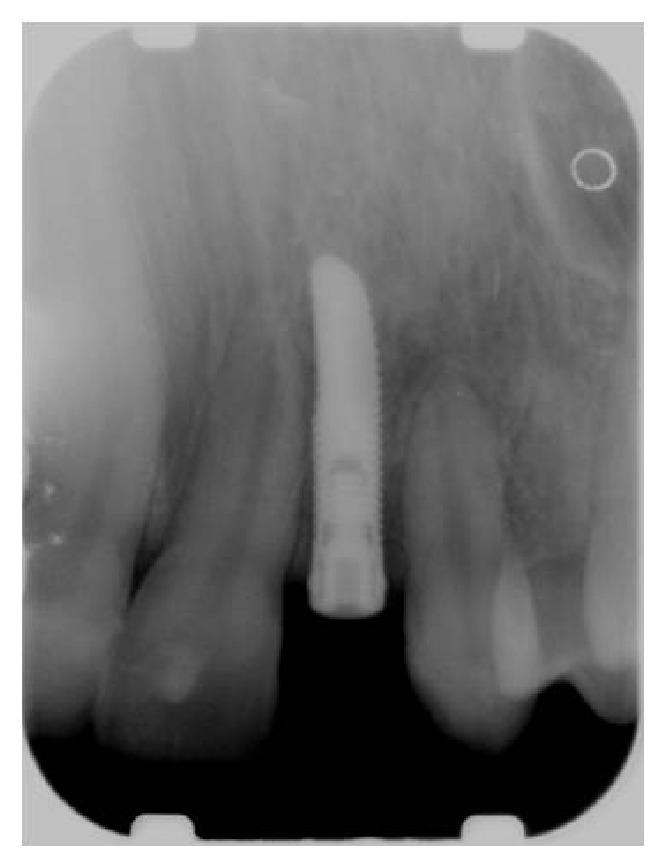
Radiographic control after implant placement in site to the right.

Soon after the insertion of the first guiding bur, an X-ray examination verified the correct inclination and position of the bur inside the bone (Figures 7, 8, and 9). After having inserted the dental implant (Figures 10 and 11) with size of 3.4 × 10.0 mm (Intra-Lock® International, Boca Raton, USA), we performed testing W-rays (Figures 12 and 13). The bone operculum was finally repositioned in the right position and the flap was properly closed with single stitches linking the periosteum.

**Figure 14:**
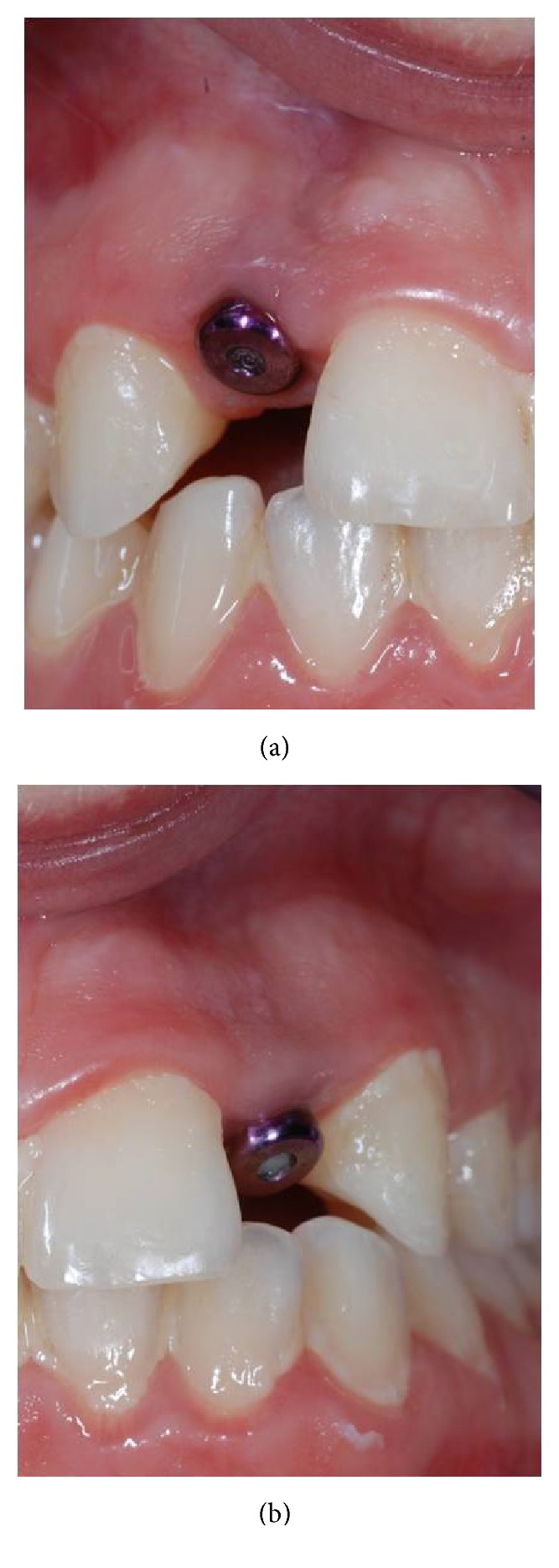
Clinical control after three months.

**Figure 15:**
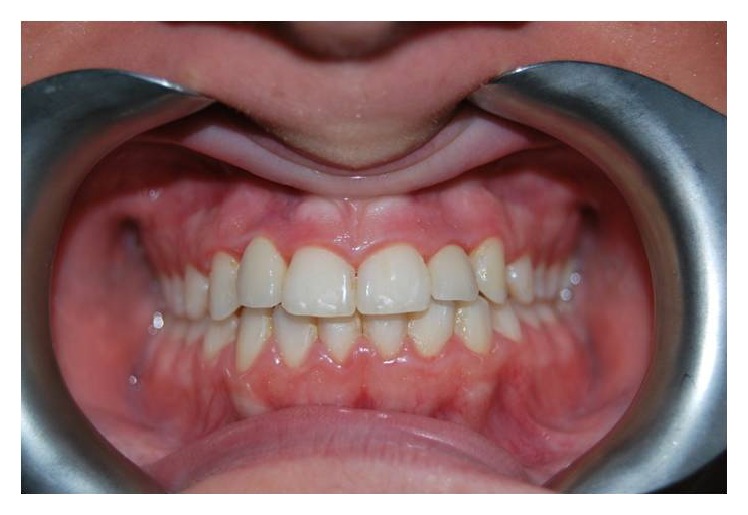
Implant prosthesization.

**Figure 16:**
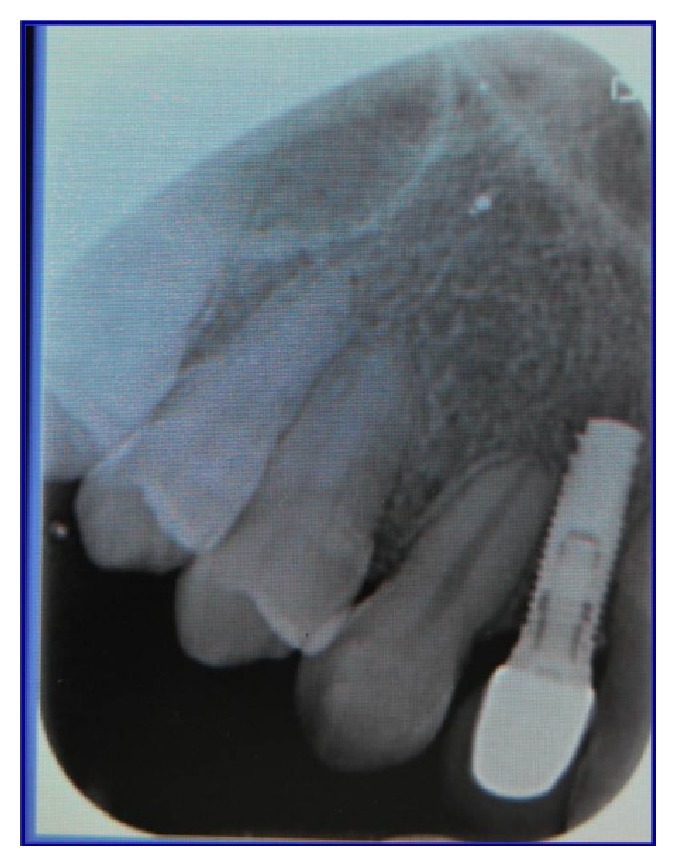
Radiographic control after one year in site to the left.

**Figure 17:**
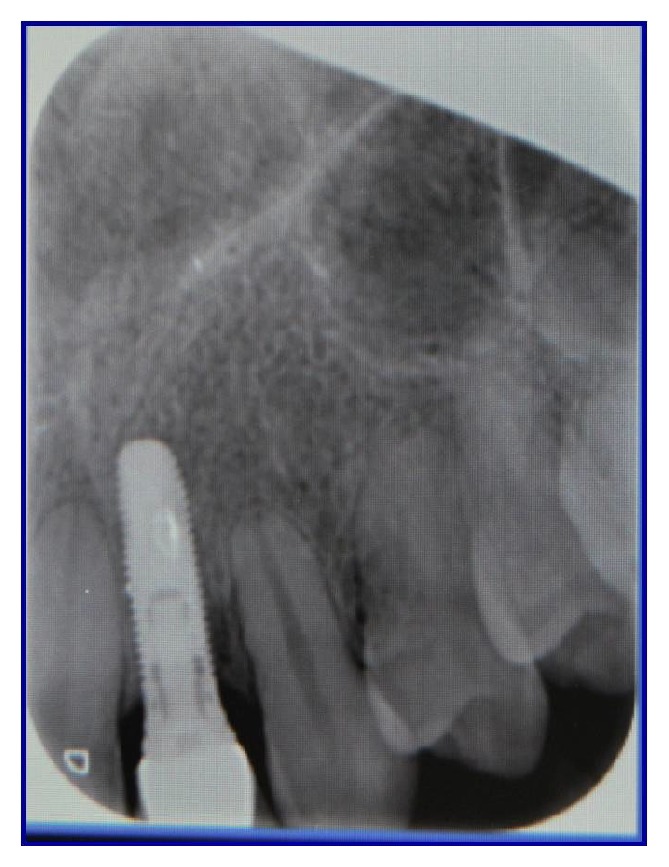
Radiographic control after one year in site to the right.

Three months later, the abutments and the crowns were added (Figures 14(a), 14(b), and 15). The next check was done one year after the prosthesization (Figures 16 and 17).

## 8. Discussion

The choice of the surgical approach detailed above was due to functional and especially esthetic needs concerning the young age of the patient.

The orthodontic treatment has led to obtaining the necessary space where the dental implants will be placed; later an orthodontic appliance of Maryland type has been placed, to ensure acceptable stability, function, and aesthetics, as clearly demonstrated in the scientific literature [11]. Reaching a suitable age, the treatment changed to an implant-prosthetics one, even with the difficulties represented by the lacking of vestibular-palatal bone depth and by the small mesial-distal space.

We know indeed that the distance between the implant and the center of the adjacent teeth should be about 7-8 mm.

A proper position towards the mesial-distal way enables the formation of an adequate contact-point.

The implant should be placed along the oral-lingual direction 1 mm behind the buccal bone in order to obtain a correct distribution of the interdental soft tissues.

The use of this procedure has been allowed to expand not only the dimension of the crest along vestibular-palatal direction but also an increased control on the implant insertion, improving the prognosis.

Thanks to the split crest approach we also obtained an emergence profile resulting as appropriate to the high esthetic requirements, with a good prosthetic rehabilitation.
